# Change of oral microbiome diversity by smoking across different age groups

**DOI:** 10.3389/fmicb.2025.1714229

**Published:** 2025-12-19

**Authors:** Kang Seo, Jin-Young Min, Kyoung-Bok Min, Kun-Hee Oh, Seung-Woo Ryoo, Seok-Yoon Son, Ji-Hyeon Lee

**Affiliations:** 1 Department of Preventive Medicine, Seoul National University College of Medicine, Seoul, Republic of Korea; 2 Veterans Medical Research Institute, Veterans Health Service Medical Center, Seoul, Republic of Korea; 3 Integrated Major in Innovative Medical Science, Seoul National University Graduate School, Jongno-gu, Seoul, Republic of Korea; 4 Institute of Environmental Medicine, Seoul National University Medical Research Center, Seoul, Republic of Korea

**Keywords:** oral microbiome, age specific, cotinine, smoking, alpha diversity, beta diversity

## Abstract

**Introduction:**

The oral microbiome, a complex ecosystem linked to both oral and systemic diseases, undergoes compositional and functional changes with aging. Tobacco exposure is a known disruptor of microbial homeostasis, yet its effect on microbial diversity remains inconsistent. Whether agingmodifies the relationship between smoking and the oral microbiome remains unclear. This study aimed to evaluate (1) the association between serum cotinine and oral microbial diversity, (2) whether this association varies by age, and (3) taxonomic shifts that may explain smoking-related dysbiosis.

**Method:**

We analyzed data from 4,387 adults aged 30–69 years in the U.S. National Health and Nutrition Examination Survey 2009–2012. Serumcotinine, an objective biomarker of nicotine exposure, was used as the primary exposure. Oral microbiome diversity was assessed via *16S rRNA* gene sequencing of oral rinse samples. Microbial profiles were analyzed using observed amplicon sequence variants and Bray-Curtis. Alpha diversity declined progressively with age, with the most pronounced reduction among current smokers.

**Results:**

Serum cotinine was inversely associated with alpha diversity, particularly in current smokers aged 60–69 years (adjusted β = −0.1081, *p* = 0.0002). Beta diversity differed significantly by smoking status (PERMANOVA *p* < 0.0001). Analysis identified 29 genera were associated with serum cotinine: *Haemophilus, Neisseria*, and *Gemella* decreased with higher exposure, while *Atopobium* and *Lactobacillus* increased. Tobacco exposure is associated with reduced oral microbial diversity, particularly in older adults.

**Discussion:**

This highlights the synergistic impact of aging and smoking on the oral microbiome and underscores the need for age-specific prevention strategies. Prospective studies are warranted to confirm causality and assess the reversibility of smoking-induced dysbiosis.

## Introduction

The oral cavity harbors one of the most diverse microbial communities in the human body—second only to the gut (Deo and Deshmukh, 2019). It contains more than 700 bacterial species, along with fungi, viruses, and archaea (Consortium, 2012; Hou et al., 2025). This complex ecosystem regulates nutrient metabolism, supports immune function, and protects against pathogen colonization (Tian et al., 2024). Beyond oral health, the oral microbiome is increasingly recognized as a contributor to systemic conditions, including cardiovascular disease, diabetes mellitus, cancer, and all-cause mortality (Irfan et al., 2020; Li et al., 2022; Tian et al., 2024; Yang et al., 2024).

Microbial diversity in the oral cavity is shaped by both intrinsic and extrinsic factors. Among intrinsic factors, aging drives a shift in microbial composition. Older adults tend to exhibit a decline in beneficial commensals and an increase in opportunistic or pro-inflammatory taxa (Chaturvedi et al., 2024). These changes may contribute to the higher burden of oral and systemic disease observed with aging (Corrêa et al., 2025; Kazarina et al., 2023).

Tobacco exposure represents a major extrinsic modifier. It introduces over 7,000 toxicants into the oral cavity, altering oxygen tension, pH, and temperature, while inducing oxidative stress (Senaratne et al., 2024). Smoking has been associated with markedly increased risks of periodontitis and oral cancer—up to 85% and 3.6-fold higher, respectively—compared to non-smokers (Noggle et al., 2024; Shapiro et al., 2022). Smoking-induced microbial dysbiosis is thought to play a mechanistic role in this relationship, promoting pathogen-dominated communities over health-associated taxa.

Yet despite this biological plausibility, the impact of smoking on oral microbiome diversity remains unresolved. Some studies report reduced alpha diversity in smokers, consistent with dysbiosis. Others, paradoxically, report higher or unchanged diversity (Huang et al., 2023; Maki et al., 2022). These inconsistencies may reflect methodological limitations. Key issues include reliance on self-reported smoking status, inadequate adjustment for oral inflammation, and failure to account for age-related biological differences (Senaratne et al., 2024).

Aging may act as a biological modifier in this context. Declines in immune surveillance, epithelial barrier function, and salivary antimicrobial capacity may reduce microbial resilience in older individuals (Kazarina et al., 2023). These vulnerabilities could amplify the disruptive effects of smoking on the oral microbiome. However, few large-scale studies have explicitly examined the interaction between age and tobacco exposure.

To address these gaps, we analyzed nationally representative data from the U.S. National Health and Nutrition Examination Survey (NHANES). We used serum cotinine as an objective biomarker of recent nicotine exposure to avoid bias from self-reported smoking. We also adjusted for clinically assessed periodontitis to isolate microbial changes attributable to tobacco exposure, independent of oral inflammation.

Our aim was twofold. First, we sought to evaluate the association between serum cotinine and oral microbial diversity across age groups. Second, we examined whether age modifies this relationship. In addition, we explored taxa-level shifts that might offer mechanistic insights into smoking-associated changes in microbial diversity.

## Materials and methods

### Data and study population

This study utilized publicly available data from NHANES, a cross-sectional, nationally representative program that assesses the health and nutritional status of the civilian, non-institutionalized U.S. population. NHANES is conducted by the National Center for Health Statistics, part of the Centers for Disease Control and Prevention (CDC), using a complex multistage probability sampling design. All survey protocols were approved by the CDC Institutional Review Board, and written informed consent was obtained from all participants. Detailed descriptions of survey methodology and procedures are available on the NHANES website (https://wwwn.cdc.gov/nchs/nhanes/).

For this analysis, we used data from the 2009–2012 NHANES cycles, the two cycles for which oral microbiome sequencing data are currently available. Of the 20,293 individuals who participated in these cycles, we first excluded participants without oral microbiome profiles (*n* = 10,945). We further excluded those who lacked serum cotinine measurements or key covariates (*n* = 4,768) and those who self-identified as never or former smokers but had serum cotinine levels >14 ng/mL (*n* = 193). The cotinine-based exclusion was to minimize misclassification due to inconsistent self-reporting and biomarker data; based on prior evidence, serum cotinine >14 ng/mL is indicative of active smoking, even among those who self-report as never or former smokers (Beghini et al., 2019). After applying these criteria, a total of 4,387 participants with complete oral microbiome data, serum cotinine measurements, and relevant covariates were included in the final analytic sample. A flow diagram illustrating participant selection is presented in Supplementary Figure S1.

### Smoking status and serum cotinine measurement

Smoking status was determined using both self-reported questionnaire responses and serum cotinine concentrations. Participants were classified as never, former, or current smokers based on their responses to the NHANES smoking questionnaire. Never smokers were defined as individuals who answered “no” to the question “Have you smoked at least 100 cigarettes in your entire life?” Former smokers were those who answered “yes” to the same question and “not at all” to the question “Do you now smoke cigarettes?” Current smokers were defined as those who answered “yes” to both items.

Serum cotinine—a primary metabolite of nicotine with a half-life of 15–20 h—was used as an objective biomarker of recent tobacco exposure. Serum specimens were collected, stored at −20 °C, and shipped under frozen conditions to the Division of Laboratory Sciences at the National Center for Environmental Health, CDC. Cotinine concentrations were measured using isotope dilution high-performance liquid chromatography coupled with atmospheric pressure chemical ionization tandem mass spectrometry (ID HPLC–APCI MS/MS). Quality assurance and control procedures followed the protocols outlined in the NHANES Laboratory Procedures Manual (https://wwwn.cdc.gov/nchs/data/nhanes/public/2009/labmethods/COT_F_met.pdf).

### Measurements of the oral microbiome

Oral microbiome data for participants aged 14–69 years (2009–2010 and 2011–2012 cycles) were obtained from the NHANES public-use data files. The data were pre-processed by NHANES, and we summarize their methodology here for clarity.

Oral rinse samples were collected by instructing participants to swish 10 mL of mouthwash for 5 seconds, gargle three times for 5 seconds each, and then expectorate into a sterile specimen container. Sample processing was conducted at the Knight Laboratory, University of California San Diego.

According to the NHANES documentation (https://wwwn.cdc.gov/nchs/data/nhanes/omp/OralMicrobiomeDataDocumentation-508.pdf), microbial DNA was extracted, and the V4 region of the 16S rRNA gene was amplified using primers 515F and 806R. Sequencing was performed on the Illumina HiSeq 2500 platform with 2 × 125 bp paired-end reads. Raw sequence data were demultiplexed using QIIME 1, and amplicon sequence variants (ASV) were inferred using the DADA2 pipeline. Taxonomic assignment was conducted using the SILVA v123 reference database. One ASV (SV1032), identified as a non-bacterial taxon, was excluded from downstream analyses. The resulting data provided by NHANES included the ASV feature table, relative abundances, and raw read counts multiple taxonomic levels (from phylum to genus). Microbial diversity metrics were also pre-calculated by NHANES. Alpha diversity indices (Observed ASVs, Faith's Phylogenetic Diversity, Shannon–Weiner, and inverse Simpson) and beta diversity metrics (Bray–Curtis dissimilarity, unweighted UniFrac, and weighted UniFrac) were calculated after 10,000 reads per sample.

This study focused on the following pre-calculated data: Observed ASVs for alpha diversity, Bray-Curtis dissimilarity for beta diversity, and genus-level taxonomic comparisons. Observed ASVs were selected as the primary alpha diversity metric to quantify taxonomic richness, as it directly reflects the number of distinct taxa per sample without phylogenetic weighting. Bray-Curtis dissimilarity was chosen for beta diversity analysis because it is based on the relative abundance of taxa, making it ideal for detecting shifts in overall community structure due to external exposures such as tobacco use.

### Covariates

Covariates were selected from NHANES data files based on their established or hypothesized associations with smoking, oral microbiome composition, or both. These included sociodemographic factors (age, sex, race/ethnicity, education, and income), lifestyle behaviors (alcohol consumption and moderate physical activity), clinical conditions (diabetes mellitus, hypertension, dyslipidemia), anthropometric measures (body mass index, BMI), and oral health status (periodontitis).

Age was categorized into four strata: 30–39, 40–49, 50–59, and 60–69 years. Race/ethnicity was classified as Mexican American, Other Hispanic, Non-Hispanic White, Non-Hispanic Black, or Other Race. Education level was categorized based on self-reported highest level of education attained: less than high school, high school graduate or equivalent, and college or above. Income was represented by the family poverty-to-income ratio (PIR), which is the ratio of family income to the poverty threshold specific to family size, as defined by the U.S. Department of Health and Human Services (https://aspe.hhs.gov/topics/poverty-economic-mobility/poverty-guidelines). PIR values below 1.0 indicate incomes below the poverty line, while higher values indicate increasing income levels relative to poverty status.

Alcohol consumption was defined by self-report of consuming ≥12 alcoholic drinks per year. Moderate physical activity was identified as a positive response to the question, “Does your work involve moderate-intensity activity that causes small increases in breathing or heart rate, such as brisk walking or carrying light loads, for at least 10 min continuously?”

Diabetes mellitus was defined as having any of the following: fasting glucose ≥126 mg/dL, HbA1c ≥6.5%, or current use of oral hypoglycemic agents or insulin. Hypertension was defined as having systolic blood pressure ≥140 mmHg or diastolic blood pressure ≥90 mmHg on two or more occasions, or self-reported use of antihypertensive medication. Dyslipidemia was defined as having LDL cholesterol ≥160 mg/dL or HDL cholesterol < 40 mg/dL (men) or < 50 mg/dL (women), or a self-reported diagnosis of high cholesterol. BMI was dichotomized into obese (BMI ≥ 30 kg/m^2^) and non-obese (BMI < 30 kg/m^2^).

Periodontitis was assessed by clinical oral examination among adults aged 30–69 years. Classification was based on clinical attachment loss (AL) and probing depth (PD) criteria. Severe periodontitis was defined as the presence of ≥2 interproximal sites with AL ≥6 mm (not on the same tooth) and ≥1 interproximal site with PD ≥5 mm. Moderate periodontitis was defined as ≥2 interproximal sites with AL ≥4 mm (not on the same tooth) or ≥2 interproximal sites with PD ≥5 mm. Mild periodontitis was defined as ≥2 interproximal sites with AL ≥3 mm and ≥2 interproximal sites with PD ≥4 mm (not on the same tooth), or one site with PD ≥5 mm. Participants with mild or moderate disease were grouped together as “other periodontitis” in the analysis (Eke et al., 2015).

### Statistical analysis

Data from the 2009–2010 and 2011–2012 NHANES cycles were combined for analysis. To account for the complex survey design and ensure nationally representative estimates, 2-year Mobile Examination Center sampling weights were applied in all descriptive and inferential analyses.

**Figure 1:**
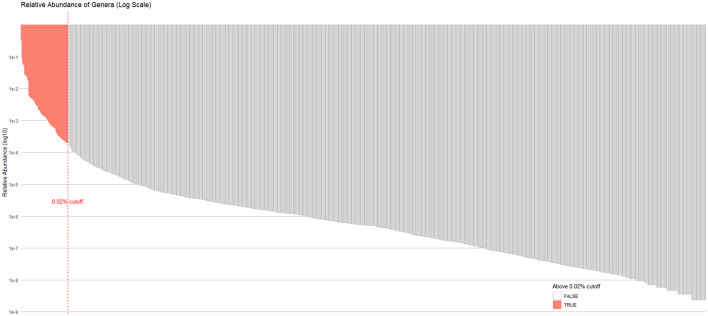
Mean relative abundance of all detected genera (*n* = 1,348) on a log_10_ scale. The bar plot displays the mean relative abundance of each genus across the entire study population. Genera are ranked in descending order of abundance. A red dashed vertical line marks the 0.02% relative abundance threshold. Genera exceeding this threshold (in red) were retained for subsequent taxonomic-level association testing, while those below were excluded to reduce noise from spurious low-abundance signals.

Categorical variables were summarized as percentages. Serum cotinine levels were compared across baseline characteristics using Student's *t*-tests for two groups, or one-way analysis of variance for three or more groups. Associations between serum cotinine concentrations and alpha diversity indices were examined using both univariate and multivariable linear regression models. The models were adjusted for demographic and clinical covariates (age, sex, race/ethnicity, body mass index, alcohol intake, and diabetes status). Beta diversity was visualized using principal coordinates analysis (PCoA) based on pairwise distance matrices with the vegan package in R. Group-level differences in microbial community composition were tested using permutational multivariate analysis of variance (PERMANOVA) with 9,999 permutations. We utilized a marginal test framework, which allows for the evaluation of the independent contribution of each variable while controlling for all others. Associations between serum cotinine and genus-level relative abundance were evaluated using linear regression models, both unadjusted and adjusted for covariates. Taxa-level analyses were restricted to genera with a mean relative abundance >0.02% (Figure 1; *n* = 89 genera). To correct for multiple testing, a Bonferroni-adjusted significance threshold of *p* < 0.000562 (0.05/89) was applied. To assess the robustness of differential abundance findings, we performed a sensitivity analysis using DESeq2, a negative binomial model analysis for count-based compositional data. DESeq2 was applied with identical covariate adjustments. Library size normalization was performed using the median-of-ratios method, and significance was determined using FDR < 0.05 (Benjamini-Hochberg correction).

All analyses were performed using R version 4.4.1 (R Foundation for Statistical Computing, Vienna, Austria). Two-sided *p*-values less than 0.05 were considered statistically significant unless otherwise noted.

## Results

### Baseline characteristics of the study population

Baseline demographic and clinical characteristics, along with serum cotinine levels, are summarized in Table 1. A total of 4,387 participants were included, comprising 2,381 never smokers, 973 former smokers, and 1,033 current smokers. The mean age was 48.8 ± 11.3 years. The participants were nearly balanced by sex (50.5% male, 49.5% female), and 41% identified as non-Hispanic White. Approximately 55% had attained a college education or higher. The prevalence of obesity, diabetes, hypertension, and dyslipidemia was 40.9%, 16.4%, 37.5%, and 57.3%, respectively. Clinically diagnosed periodontitis was present in 36.1% of participants, with 23.3% classified as mild to moderate and 12.8% classified as severe. The mean serum cotinine concentration was 54.5 ± 119.6 ng/mL, reflecting varying degrees of tobacco exposure. Notably, serum cotinine levels differed significantly across sex, age categories, race/ethnicity, educational attainment, household income, BMI, alcohol consumption, physical activity, diabetes, periodontitis status, and smoking status.

**Table 1:** Characteristics of the study population by serum cotinine levels.

**Characteristics**	**N (%)**	**Serum cotinine (ng/mL)**	***p*-value^1^**
Total	4,387	54.5 ± 119.6	
**Sex**			**< 0.0001**
Male	2,215 (50.5)	64.9 ± 128.4	
Female	2,172 (49.5)	44.0 ± 108.8	
**Age, y**	**48.8** **±11.3**		**0.0014**
30–39	1,138 (25.9)	55.4 ± 115.3	
40–49	1,163 (26.5)	61.2 ± 124.9	
50–59	1,037 (23.6)	58.3 ± 125.7	
60–69	1,049 (23.9)	42.4 ± 110.7	
**Ethnicity**			**< 0.0001**
Mexican American	704 (16.0)	22.0 ± 68.4	
Other Hispanic	458 (10.4)	25.9 ± 81.4	
Non-Hispanic White	1,794 (40.9)	71.1 ± 131.8	
Non-Hispanic Black	990 (22.6)	71.9 ± 141.7	
Other race	441 (10.1)	29.8 ± 85.1	
**Education**			**< 0.0001**
Less than high school	1,045 (23.8)	79.5 ± 140.1	
High school	940 (21.4)	73.9 ± 134.3	
College or above	2,402 (54.8)	36.1 ± 98.8	
**Family income (PIR)** ^2^			**< 0.0001**
≤ 1.3	1,387 (31.6)	92.7 ± 147.2	
>1.3, ≤ 3.5	1,466 (33.4)	49.4 ± 113.9	
>3.5	1,534 (35.0)	24.9 ± 81.7	
**BMI**			**< 0.0001**
< 30	2,594 (59.1)	64.6 ± 131.5	
≥30	1,793 (40.9)	40.0 ± 98.0	
**Alcohol consumption status**			**< 0.0001**
< 12 drinks/year	1,107 (25.2)	26.9 ± 90.8	
≥12 drinks/year	3,280 (74.8)	63.9 ± 126.5	
**Physical activity**			**0.0009**
No	2,807 (64.0)	49.9 ± 114.7	
Moderate work activity	1,580 (36.0)	62.7 ± 127.4	
**Diabetes mellitus**			**0.0204**
No	3,669 (83.6)	56.3 ± 121.0	
Yes	718 (16.4)	45.5 ± 111.7	
**Hypertension**			0.6567
No	2,741 (62.5)	53.9 ± 118.8	
Yes	1,646 (37.5)	55.6 ± 120.7	
**Dyslipidemia**			0.2695
No	1,874 (42.7)	52.2 ± 118.2	
Yes	2,513 (57.3)	56.2 ± 120.6	
**Periodontitis**			**< 0.0001**
No	2,804 (63.9)	43.5 ± 108.2	
Other periodontitis	1,022 (23.3)	61.0 ± 120.2	
Severe periodontitis	561 (12.8)	97.9 ± 156.4	
**Smoking**			**< 0.0001**
Never	2,381 (54.3)	0.2 ± 1.0	
Former	973 (22.2)	0.4 ± 1.5	
Current	1,033 (23.5)	230.7 ± 141.7	

Values are presented as number (%) for categorical variables and mean ± standard deviation (SD) for continuous variables. *Min*, minute; *PIR*, Family poverty to income ratio. ^1^*p*-value is calculated by the Student *t-*test or Analysis of variance (ANOVA) for each covariate. ^2^Family income is expressed as the ratio of family income to poverty (PIR), according to the Department of Health and Human Services (HHS) poverty guidelines.

### Alpha diversity by smoking status and age

**Figure 2:**
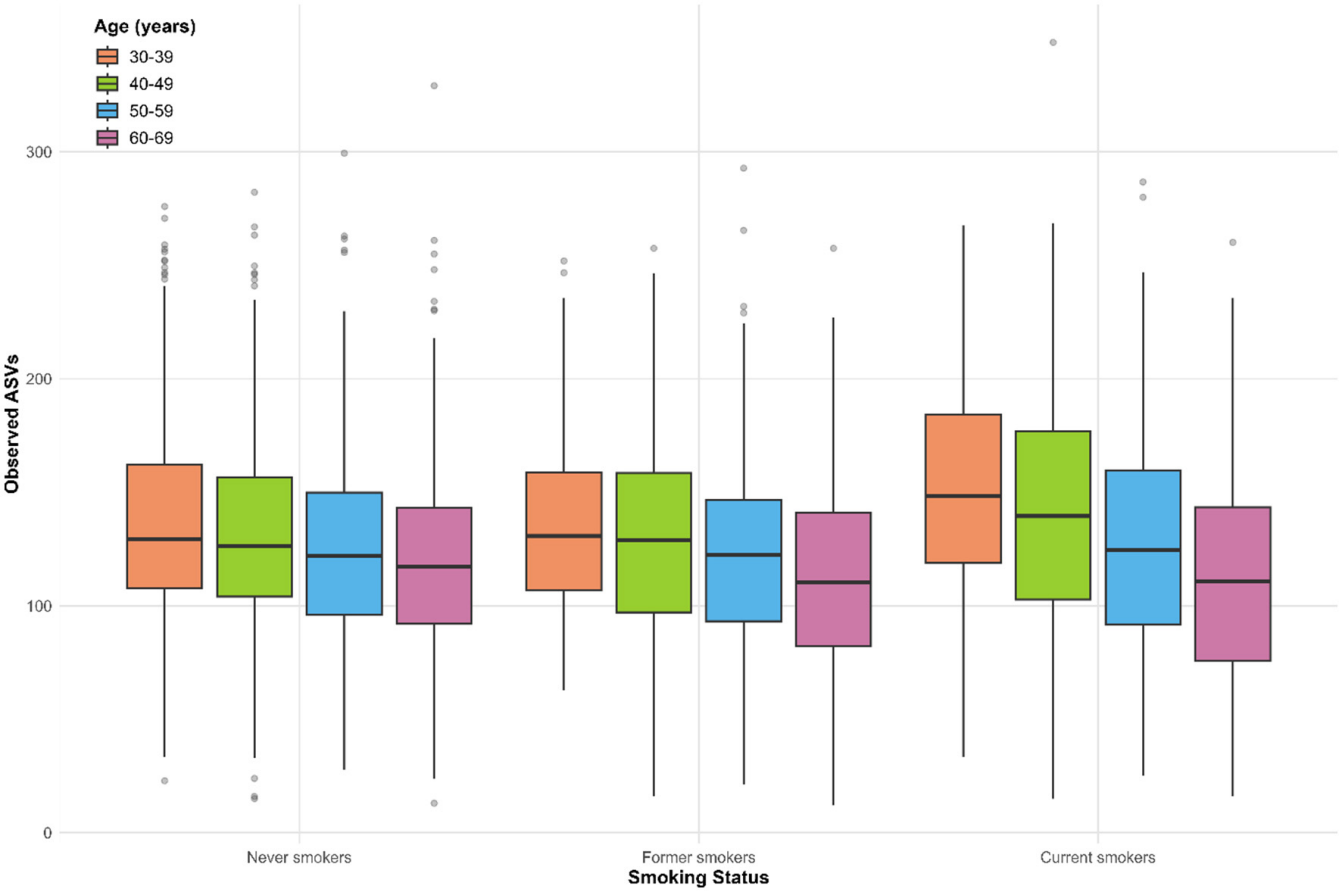
Observed ASVs (alpha diversity) by smoking status and age group. Boxplots represent the distribution of observed ASV counts (richness) across age categories (30–39, 40–49, 50–59, 60–69 years), stratified by smoking status (never, former, and current smokers). The center line of each box indicates the median, the box edges represent the interquartile range (IQR), and the whiskers extend to 1.5 × IQR. Individual dots represent outliers beyond this range.

Figure 2 illustrates the alpha diversity of the oral microbiome among never, former, and current smokers across different age groups, as measured by ASV richness. The mean ASV count among the study population was 129.0 ± 44.0. A progressive decline in alpha diversity with increasing age was observed in both former and current smokers, with the most pronounced reduction noted among current smokers. In younger age groups (30–39 and 40–49 years), current smokers exhibited higher alpha diversity compared to never smokers, whereas this trend was not seen in older age groups (50–59 and 60–69 years). These findings demonstrate that the relationship between smoking and alpha diversity varies by age group.

### Association between serum cotinine and alpha diversity

The associations between serum cotinine levels and oral microbiome alpha diversity across different age groups are presented in Table 2. In the combined analysis of never, former and current smokers, serum cotinine concentration showed a statistically significant inverse association with oral microbiome alpha diversity after multivariable adjustment in the overall sample (β = −0.0240; SE = 0.0081; *p* = 0.0082). Age-stratified models revealed the strongest inverse association among participants aged 60–69 years, both in crude (β = −0.0446; SE = 0.0167; *p* = 0.0119) and adjusted (β = −0.0719; SE = 0.0142; *p* = 0.0001) models. No significant associations were detected in younger age groups after adjustment, although a positive trend was noted in those aged 30–39 (β = 0.0250; *p* = 0.1225). In the analysis limited to former and current smokers, the overall association remained significant after adjustment (β = −0.0209; SE = 0.0082; *p* = 0.0201), with the strongest effect observed in the 60–69 age group (β = −0.0549; SE = 0.0158; *p* = 0.0028). Among current smokers only, the negative association between serum cotinine and alpha diversity was more pronounced. The association was still significant in the total current smoking population after adjustment (β = −0.0339; SE = 0.0117; *p* = 0.0098), strong and statistically significant inverse associations were observed in the 40–49 (β = −0.0524; SE = 0.0238; *p* =0.0437) and 60–69 (β = −0.1081; SE = 0.0216; *p* = 0.0002) age groups.

**Table 2:** Estimated β coefficients (SE) from linear regression models assessing the association between serum cotinine levels and oral microbiome alpha diversity (observed ASVs), stratified by age group.

**Age, y**	**N**	**Never, former and current (*****N =*** **4,387)**			
		**Crude**		**Adjusted** ^1^	
		β **(SE)**	***p*** **-value**	β **(SE)**	***p*** **-value**
Unstratified	4,387	0.0180 (0.0082)	**0.0351**	−0.0240 (0.0081)	**0.0082**
30–39	1,138	0.0603 (0.0162)	**0.0008**	0.0250 (0.0154)	0.1225
40–49	1,163	0.0243 (0.0144)	0.1018	−0.0306 (0.0153)	0.0610
50–59	1,037	0.0002 (0.0173)	0.9921	−0.0311 (0.0176)	0.0942
60–69	1,049	−0.0446 (0.0167)	**0.0119**	−0.0719 (0.0142)	**0.0001**
46.2-1.0,15.5		**Former and Current (*****N** =* **2,006)**			
		**Crude**		**Adjusted** ^1^	
		β **(SE)**	***p-*** **value**	β **(SE)**	***p*** **-value**
Unstratified	2006	0.0176 (0.0089)	0.0577	−0.0209 (0.0082)	**0.0201**
30–39	463	0.0437 (0.0188)	**0.0277**	0.0178 (0.0170)	0.3125
40–49	500	0.0115 (0.0148)	0.4419	−0.0293 (0.0161)	0.0861
50–59	418	−0.0002 (0.0182)	0.9918	−0.0191 (0.0176)	0.2927
60–69	525	−0.0364 (0.0173)	**0.0431**	−0.0549 (0.0158)	**0.0028**
46.2-1.0,15.5		**Current only (*****N** =* **1,033)**			
		**Crude**		**Adjusted** ^1^	
		β **(SE)**	***p*** **-value**	β **(SE)**	***p-*** **value**
Unstratified	1,033	−0.0339 (0.0110)	**0.0044**	−0.0339 (0.0117)	**0.0098**
30–39	296	−0.0081 (0.0253)	0.7522	−0.0104 (0.0251)	0.6868
40–49	307	−0.0264 (0.0221)	0.2416	−0.0524 (0.0238)	**0.0437**
50–59	246	−0.0061 (0.0298)	0.8404	0.0008 (0.0226)	0.9711
60–69	184	−0.0717 (0.0302)	**0.0250**	−0.1081 (0.0216)	**0.0002**

Values are presented as beta (standard error, SE). N is the number of subjects for each stratum. ^1^Adjusted for age, sex, race/ethnicity, education, family income (PIR), BMI, alcohol consumption, physical activity, diabetes mellitus, hypertension, dyslipidemia, and periodontitis.

Similar trends were observed across alternative alpha diversity indices, as shown in Supplementary Table S1. In the adjusted models for current smokers, the association remained statistically significant for Faith's phylogenetic diversity and the Shannon-Weiner index, but was not significant for the inverse Simpson index. However, among those aged 60–69, the negative association with serum cotinine was consistent across all three diversity metrics —Faith's phylogenetic diversity, Shannon-Weiner index, and inverse Simpson index—in the adjusted models. These patterns were also largely retained in the combined analysis including never smokers, suggesting robustness across analytic approaches and study populations.

### Beta diversity by smoking status

**Figure 3:**
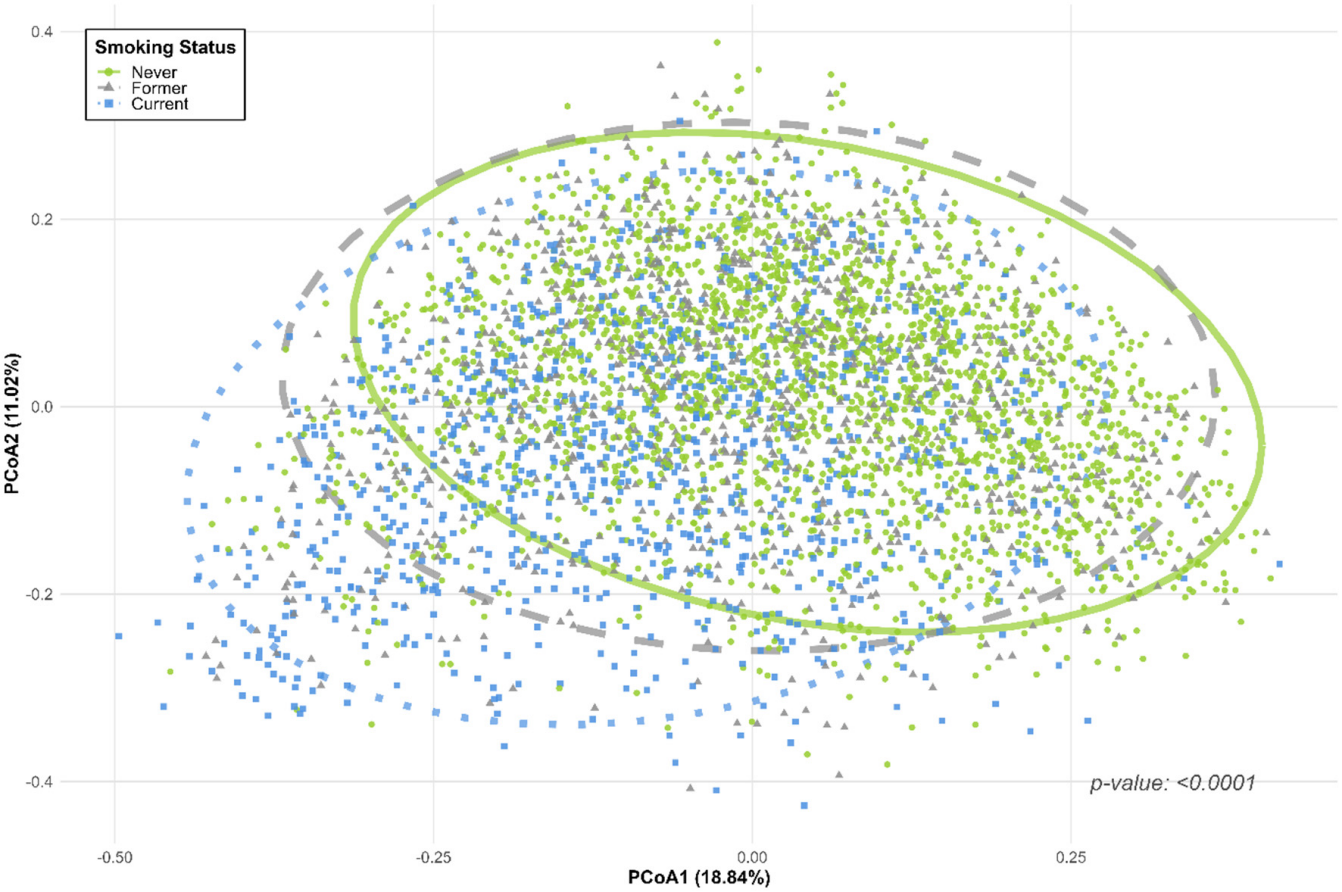
Principal coordinates analysis (PCoA) of oral microbiome beta diversity based on Bray-Curtis dissimilarity by smoking status. Each point represents the microbial community of an individual, with 95% confidence ellipses for never smokers (green circles, *n* = 2,381; solid line), former smokers (gray triangles, *n* = 973; dashed line), and current smokers (blue squares, *n* = 1,033; dotted line). PERMANOVA revealed significant differences in microbial community composition between smoking status groups (R^2^ = 0.0238, *p* < 0.0001), after adjusting for age, sex, education, income, BMI, alcohol, physical activity, diabetes, hypertension, dyslipidemia, and periodontitis.

Figure 3 illustrates the beta diversity of the oral microbiome among never, former and current smokers based on Bray-Curtis dissimilarity using PCoA. A statistically significant shift between the three groups was observed in the ordination space (PERMANOVA R2 = 0.0238, *p* < 0.0001), indicating differences in overall microbial community composition after adjusting for age, sex, education, income, BMI, alcohol, physical activity, diabetes, hypertension, dyslipidemia, and periodontitis. Although substantial overlap exists among groups, the statistically significant separation confirms that current and former smokers exhibit compositional shifts compared to never smokers.

### Genus-level shifts associated with serum cotinine

**Figure 4:**
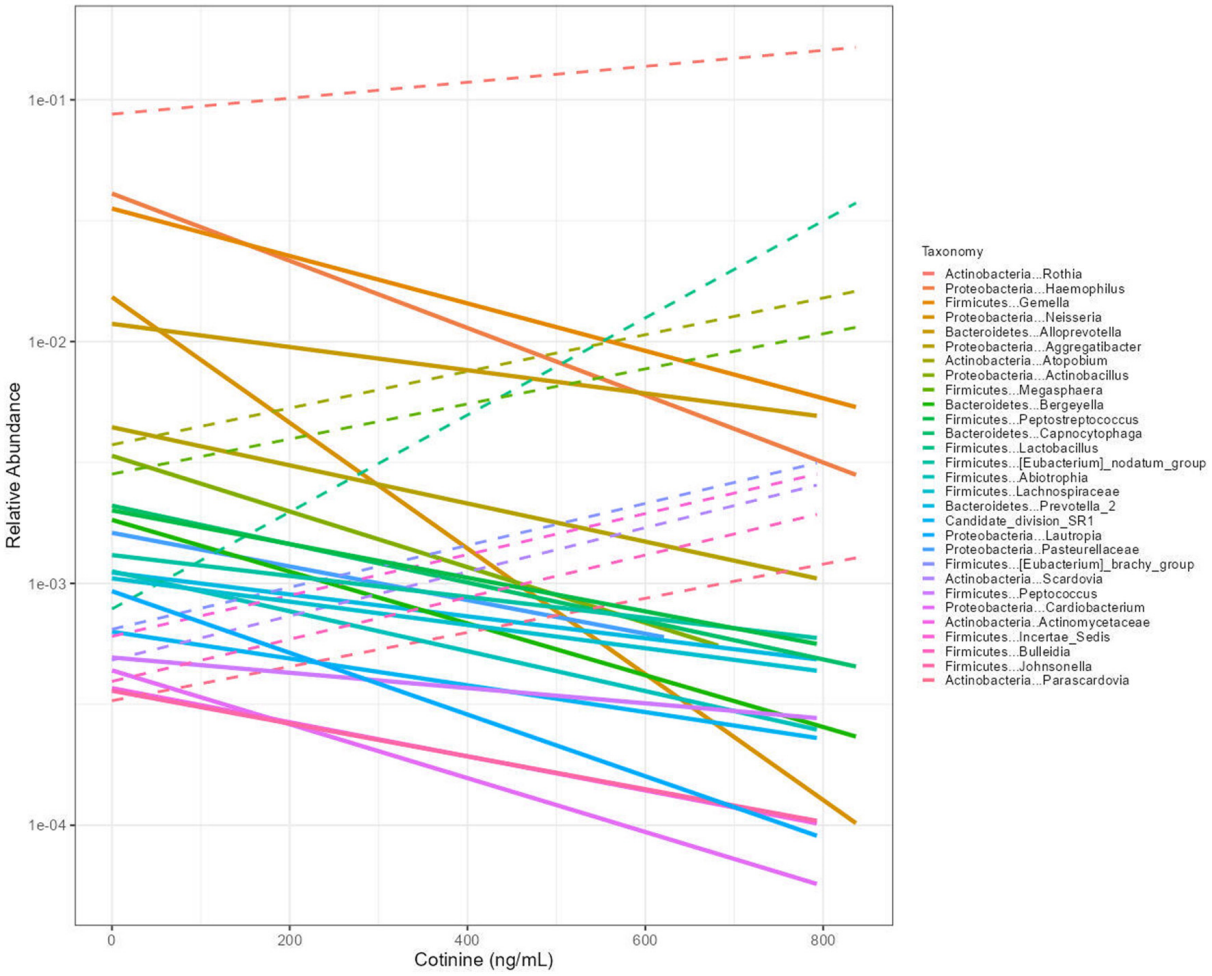
Linear trends in the relative abundance of 29 oral bacterial genera in relation to serum cotinine concentration. Each line represents a genus with a statistically significant association in the fully adjusted model (Bonferroni-corrected *p* < 0.000562). Relative abundances are shown on a log10 scale, with cotinine levels (ng/mL) on the x-axis. Genera exhibiting a decreasing abundance with increasing tobacco exposure are represented by continuous lines, while those with increasing abundance are indicated by dashed lines.

To investigate taxon-specific associations, we replaced alpha diversity with genus-level relative abundance as the outcome in fully adjusted linear regression models. A total of 29 bacterial genera showed statistically significant associations with serum cotinine levels after Bonferroni correction for multiple comparisons (*p* < 0.000562). These associations are visualized in Figure 4.

Among these, the majority displayed a negative trend, with relative abundance decreasing in association with higher cotinine levels. Genera exhibiting this decreasing pattern included *Haemophilus, Gemella, Neisseria, Alloprevotella, Aggregatibacter, Actinobacillus, Bergeyella, Peptostreptococcus, Capnocytophaga, Eubacterium nodatum* group, *Abiotrophia, Prevotella, Lautropia, Peptococcus, Cardiobacterium*, and *Johnsonella*. In contrast, nine genera demonstrated positive associations with cotinine levels, showing increasing relative abundance across the exposure gradient. These included *Rothia, Atopobium, Megasphaera, Lactobacillus, Eubacterium brachy* group, *Scardovia, Bulledia*, and *Parascardovia*. This inverse pattern, where the majority of genera decrease while a smaller subset increases with tobacco exposure, indicates selective shifts in community composition rather than uniform changes across all taxa. The differential responses of these genera highlight the heterogeneous impact of smoking on the oral microbiome at the taxonomic level.

### Sensitivity analysis using DESeq2

In sensitivity analyses using DESeq2 on raw genus counts, all 29 genera identified by linear regression models maintained the same direction of effect, and 24 of 29 (83%) also met the significance threshold of FDR < 0.05. This high concordance in both direction and statistical significance (Supplementary Table S2, Figure S2) validates the robustness of the smoking-associated genus-level compositional shifts identified in the primary analysis.

## Discussion

In this large, population-based study of never, former, and current smokers, we found that the association between tobacco exposure and oral microbial diversity was significantly modified by age. Serum cotinine concentrations were inversely associated with alpha diversity in an age-dependent manner, with the strongest effects observed among current smokers aged 60–69 years. This pattern suggests that prolonged or cumulative exposure to tobacco may disproportionately disrupt microbial ecosystems in later life, potentially reflecting both heightened biological vulnerability and diminished host–microbe homeostasis with aging.

In contrast, a recent systematic review by Senaratne et al. (2024) synthesized evidence from 36 studies and reported that tobacco users generally exhibit higher oral microbial diversity than non-users in the majority of reports (seen in 22 of 36 reports) (Senaratne et al., 2024). Only four studies reported a lower diversity in smokers (Senaratne et al., 2024). These conflicting findings underscore the ongoing debate regarding the directionality of the relationship between smoking and microbial richness. However, many of the studies included in that review were conducted predominantly in young to middle-aged adults, with few stratifying or specifically analyzing older populations. Substantial heterogeneity across studies—stemming from differences in sampling techniques, target oral niches, sequencing platforms, and definitions of tobacco exposure—further complicates interpretation. These methodological inconsistencies highlight the need for higher-quality research with standardized protocols and age-specific analyses.

Our study addresses key limitations in the existing literature by using a large, demographically diverse cohort and incorporating clinically assessed periodontitis as a covariate. Whereas prior studies often relied on small samples (22 to 1,616 participants) with narrow age ranges, our population-based design enabled unified analysis across a broad age spectrum, allowing for the identification of age-specific susceptibilities in the relationship between tobacco exposure and oral microbial diversity. We further enhanced exposure assessment by using serum cotinine as a continuous, objective biomarker that reflects both the intensity and recency of tobacco use (Pérez-Stable et al., 1995), enabling more precise modeling of dose-dependent microbial shifts.

Additionally, by adjusting for clinically assessed periodontitis—a known modifier of oral microbiome composition and a common comorbidity of smoking (Arredondo et al., 2023; Nociti et al., 2015)—we were able to isolate the independent effects of tobacco exposure. Together, these methodological strengths improve the internal validity of our findings and allow for more nuanced interpretation of host–microbiome interactions across the adult lifespan.

Our finding of a negative association between smoking and alpha diversity highlights the impact of tobacco exposure on oral microbial ecology. This association was most pronounced among current smokers aged 60–69, underscoring the role of age as a biological modifier in this relationship. The observed decline in oral microbial diversity with advancing age may be interpreted through the lens of immunological remodeling described by Fulop et al. (2017). Rather than reflecting a linear deterioration of immune function, age-related immunosenescence and inflamm-aging may represent adaptive processes that prioritize immune economy and control of known pathogens. However, such remodeling may inadvertently reduce microbial surveillance and mucosal resilience, leading to a dysbiotic and more vulnerable oral microbiome in older adults. This vulnerability is likely amplified by chronic tobacco exposure, which assaults the oral mucosa through two synergistic mechanisms. First the thousands of combustions by products (e.g., reactive oxygen species) introduce the well-documented oxidative stress (Lee et al., 2012; Tharakan et al., 2016). Second, as suggested by our use of cotinine, nicotine itself is not a neutral biomarker but an active etiological agent. Nicotine acts as a potent vasoconstrictor, reducing gingival blood flow and creating a hypoxic microenvironment that selects for anaerobic pathogens (Palmer et al., 2005). Furthermore, nicotine binds directly to nAChRs on oral epithelial and immune cells, which actively promotes mucosal inflammation and disrupts epithelial barrier integrity—the very effects vaguely attributed to smoking in the past (Zhang et al., 2022; Katheder et al., 2023). Some *in vitro* evidence even shows nicotine can directly enhance biofilm formation in pathogens like *P. gingivalis* (Hanioka et al., 2019).

This synergistic destabilization may explain why smoking alters the composition of the oral microbiome, increasing the abundance of bacteria such as *Atopobium* and *Veillonella*—which includes species associated with periodontal disease and oral inflammation—while decreasing commensal or health-associates genera like *Neisseria* and *Haemophilus* (Chattopadhyay et al., 2024; Maki et al., 2022). These compositional shifts may synergize with age-related immune decline, compounding dysbiosis and accelerating the loss of microbial diversity (Bashir et al., 2023). In individuals with age-compromised immune and barrier functions, smoking may thus act as an ecological stressor that amplifies microbiome destabilization beyond the effects of aging alone. This interaction highlights the need to consider both intrinsic (aging) and extrinsic (smoking) factors in characterizing oral microbiome dynamics in older adults. In parallel, age-related degradation of the oral mucosal barrier and salivary antimicrobial defenses may create a permissive environment for dysbiosis (Rajasekaran et al., 2024; Pei et al., 2024). These structural and biochemical vulnerabilities, when compounded by tobacco-induced oxidative and inflammatory stress, likely exacerbate the microbial diversity loss observed in older smokers (Imdad et al., 2025).

At the genus level, our analysis identified distinct patterns in the oral microbiome associated with increasing serum cotinine levels. Notably, several genera exhibited significant negative associations, including *Haemophilus, Neisseria*, and *Gemella*. *Haemophilus* and *Neisseria* are Gram-negative facultative anaerobes known to be cornerstone species in oral health. Their key role involves reducing dietary nitrate (from saliva) to nitrite, the first step in the entero-salivary nitrate-nitrite-nitric oxide pathway, which has crucial systemic vascular implications (e.g., blood pressure regulation) (Rosier et al., 2024; Yang et al., 2025). Their depletion in smokers, particularly among individuals aged 60–69, aligns with previous findings indicating reduced abundance of these genera in smokers' oral microbiota (Antonello et al., 2023). This reduction may therefore contribute not only to impaired local mucosal immunity but also to systemic endothelial dysfunction. Conversely, genera such as *Bulledia, Veillonella*, and *Atopobium* were positively associated with higher cotinine levels. These anaerobic bacteria are often linked to pro-inflammatory conditions and have been found in greater abundance in smokers (Jia et al., 2021; Wu et al., 2016). *Atopobium*, in particular, has been associated with periodontal disease and oral carcinogenesis, suggesting that its proliferation in smokers could have pathogenic consequences (Zhou et al., 2024; Perera et al., 2018). Similarly, *Veillonella* species are known for their acidogenic properties, which can contribute to dental caries development (Gross et al., 2012). *Bulledia* species, including *Bulledia moorei*, have been identified as an opportunistic pathogen enriched in tobacco users, with potential roles in oral dysbiosis and inflammatory conditions (Chattopadhyay et al., 2024). Interestingly, *Lactobacillus*-through traditionally regarded as beneficial for its probiotic properties and antagonism against pathogens-also demonstrated a positive association with cotinine levels (Huang et al., 2024; Tian et al., 2024). However, its acidogenic nature can lower oral pH, creating an environment conducive to enamel demineralization and cariogenic biofilms (Wen et al., 2022). This dual role highlights the context-dependent effects of specific taxa and the ecological complexity of the oral microbiome. Collectively, these taxonomic shifts suggest that tobacco exposure imposes selective pressures on the oral microbial community—suppressing health-associated genera while enriching anaerobic and acid-tolerant species. Such compositional reorganization may destabilize the oral ecosystem, promoting dysbiosis and increasing susceptibility to both local and systemic diseases. From a public health perspective, our findings highlight the importance of integrating oral microbiome monitoring into tobacco cessation programs, particularly for older adults who may be most vulnerable to microbiome-mediated health consequences.

Several limitations should be considered. First, the cross-sectional design of the NHANES data precludes causal inference. While the observed dose-dependent relationship between serum cotinine levels and alpha diversity supports a directional association, the biological implausibility of reverse causality (i.e., oral microbiota influencing smoking behavior) further strengthens this inference. Nonetheless, longitudinal studies are needed to confirm temporal sequencing and to rule out residual confounding. Second, although smoking status was self-reported, potential misclassification was mitigated by the concurrent use of serum cotinine. This validated biomarker objectively reflects both exposure intensity and recency (Benowitz et al., 2009), thereby improving exposure ascertainment compared to self-report alone. Third, while our models were adjusted for major known confounders (including age, sex, and periodontitis), we could not control for other potential confounders such as detailed oral hygiene behaviors, specific dietary habits, or medication use, as these data were not comprehensively available in NHANES. As these factors can independently alter oral microbial communities, future studies should more comprehensively assess and control these variables to further isolate the effect of tobacco exposure. Fourth, the study population was limited to adults aged 30–69 due to the scope of oral examination protocols within NHANES. As such, our findings may not generalize to younger or older age groups beyond this range. Fifth, our taxonomic profiling was based on *16S rRNA* gene sequencing. While this approach is robust for assessing community-level diversity and genus-level composition, it lacks the resolution needed to distinguish species-level differences or infer functional activity. Future studies employing shotgun metagenomics or metatranscriptomics could provide deeper insights into the functional consequences of smoking-related dysbiosis and reveal microbiome-derived metabolites relevant to host health.

In conclusion, this population-based analysis reveals a significant inverse association between tobacco exposure and oral microbial alpha diversity, with the strongest effects observed in older current smokers. These changes were accompanied by significant taxonomic shifts, notably the depletion of health-associated, nitrate-reducing genera like *Haemophilus* and *Neisseria* and the enrichment of pro-inflammatory anaerobes such as *Atopobium* and *Veillonella*.

From a clinical perspective, these findings highlight the compounded vulnerability of aging oral ecosystems to tobacco-related perturbations, underscoring the importance of age-tailored prevention and cessation strategies. However, as this is a cross-sectional, observational study, prospective, longitudinal research is needed to confirm causality and clarify the timeline and reversibility of smoking-induced microbial alterations.

## Data Availability

The datasets analyzed during the current study are available from the NHANES repository, https://wwwn.cdc.gov/nchs/nhanes/.
