# Supplementary material for: Change of oral microbiome diversity by smoking across different age groups
Source: Front Microbiol. 2025 Dec 19;16:1714229. doi: 10.3389/fmicb.2025.1714229 (PMC12758414; doi:10.3389/fmicb.2025.1714229)
Supplement: Supplementary file 1 [file Data_Sheet_1.pdf]

Text summary

Flowchart of participant selection and exclusion criteria for the final study population.

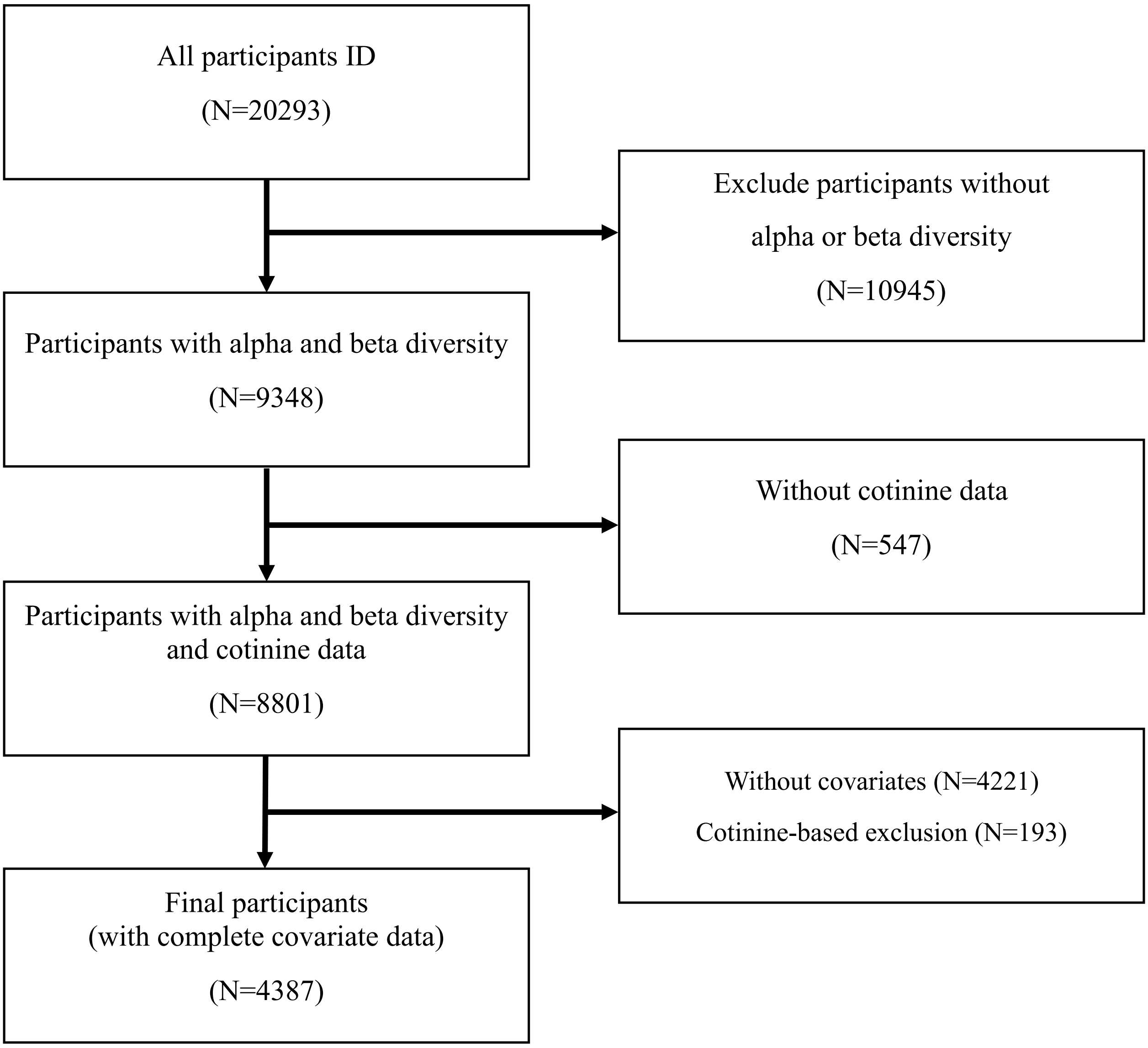

Figure S1. Flowchart of study participant selection from the NHANES dataset. Of the 20293 individuals initially identified, 9348 had available alpha and beta diversity data. After excluding those without serum cotinine data (n = 547), 8801 remained. Participants lacking covariate information (n = 4221) and those with cotinine levels over 14 ng/mL among never and former smokers (n = 193) were excluded, yielding a final analytic sample of 4387 Never, former and current smokers with complete data for diversity and exposure analysis.
