# Supplementary material for: Change of oral microbiome diversity by smoking across different age groups
Source: Front Microbiol. 2025 Dec 19;16:1714229. doi: 10.3389/fmicb.2025.1714229 (PMC12758414; doi:10.3389/fmicb.2025.1714229)
Supplement: Supplementary file 2 [file Data_Sheet_2.pdf]

Text summary

Forest plot of DESeq2 sensitivity analysis of the 29 genera identified by linear models.

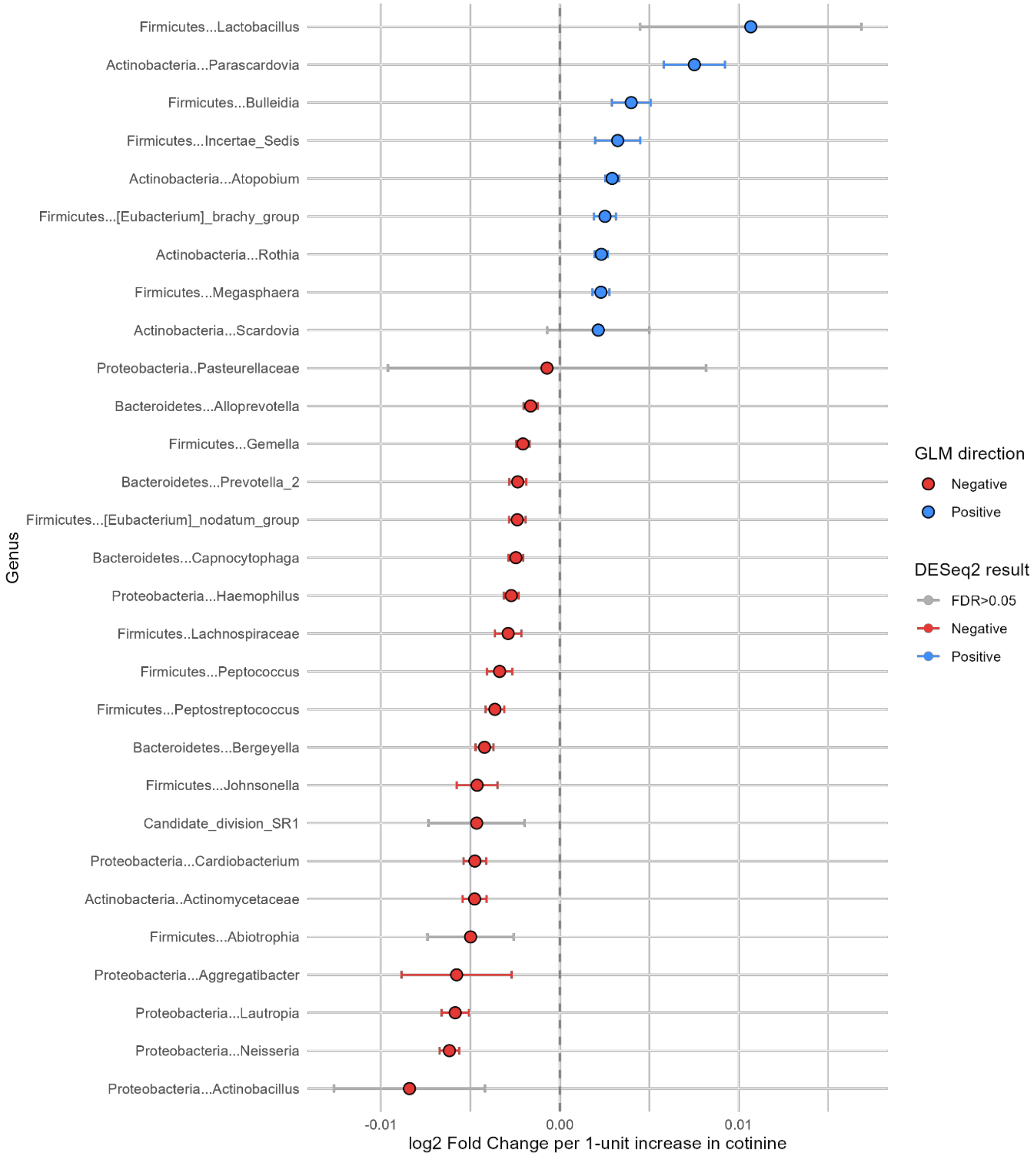

Figure S2. DESeq2 sensitivity analysis of the 29 genera identified by linear models. Forest plot of DESeq2 log2 fold-change per 1 ng/mL increase in serum cotinine with 95% Wald confidence intervals. Genera are ordered by effect size and labeled as in Figure 3. Point color indicates the direction of association from the primary linear model: blue (positive GLM association), red (negative GLM association). Confidence interval color indicates the DESeq2 result: blue(positive, FDR <0.05), red(negative, FDR <0.05), or gray (Insignificant, FDR ≥0.05).

Vertical dashed line marks zero effect. DESeq2 used median-of-ratios normalization and Benjamini–Hochberg FDR control. All 29 genera demonstrated directional concordance between GLM and DESeq2 analyses.
