# Supplementary material for: Change of oral microbiome diversity by smoking across different age groups
Source: Front Microbiol. 2025 Dec 19;16:1714229. doi: 10.3389/fmicb.2025.1714229 (PMC12758414; doi:10.3389/fmicb.2025.1714229)
Supplement: Supplementary file 3 [file Data_Sheet_3.pdf]

Text summary

Association between serum cotinine levels and alternate alpha-diversity metrics (Faith's phylogenetic diversity, Shannon-Weiner index, Inverse Simpson index), stratified by age group.

Table S1. Estimated β coefficients (SE) from linear regression models assessing the association between serum cotinine levels and oral microbiome alpha diversity metrics, stratified by age group.

Faith's phylogenetic diversity

		**Never, Former and Current**			
		**Crude**		**Adjusted[1]**	
**Age, y**	**N**	**β (SE)**	**p-value**	**β (SE)**	**p-value**
Unstratified	4387	0.0020 (0.0006)	**0.0038**	-0.0015 (0.0006)	**0.0338**
30-39	1138	0.0045 (0.0013)	**0.0012**	0.0017 (0.0012)	0.1859
40-49	1163	0.0027 (0.0012)	**0.0310**	-0.0016 (0.0013)	0.2266
50-59	1037	0.0006 (0.0014)	0.6885	-0.0021 (0.0015)	0.1713
60-69	1049	-0.0021 (0.0013)	0.1260	-0.0045 (0.0011)	**0.0009**
		**Former and Current**			
		**Crude**		**Adjusted[1]**	
**Age, y**	**N**	**β (SE)**	**p-value**	**β (SE)**	**p-value**
Unstratified	2006	0.0019 (0.0007)	**0.0109**	-0.0013 (0.0007)	0.0741
30-39	463	0.0029 (0.0015)	0.0723	0.0010 (0.0015)	0.5026
40-49	500	0.0017 (0.0014)	0.2127	-0.0014 (0.0014)	0.3358
50-59	418	0.0005 (0.0014)	0.7501	-0.0009 (0.0014)	0.5118
60-69	525	-0.0016 (0.0015)	0.2779	-0.0034 (0.0012)	**0.0140**
		**Current only**			
		**Crude**		**Adjusted[1]**	
**Age, y**	**N**	**β (SE)**	**p-value**	**β (SE)**	**p-value**
Unstratified	1033	-0.0025 (0.0009)	**0.0109**	-0.0026 (0.0011)	**0.0287**
30-39	296	-0.0007 (0.0023)	0.7720	-0.0008 (0.0023)	0.7436
40-49	307	-0.0016 (0.0018)	0.3930	-0.0035 (0.0020)	0.0952

50-59	246	-0.0009 (0.0025)	0.7206	0.0000 (0.0019)	0.9884
60-69	184	-0.0054 (0.0024)	**0.0372**	-0.0088 (0.0016)	**0.0001**

Shannnon-Weiner index

		**Never, Former and Current**			
		**Crude**		**Adjusted[1]**	
**Age, y**	**N**	**β (SE)**	**p-value**	**β (SE)**	**p-value**
Unstratified	4387	0.0000 (0.0001)	0.8020	-0.0005 (0.0001)	**0.0019**
30-39	1138	0.0004 (0.0003)	0.1008	0.0001 (0.0003)	0.7730
40-49	1163	0.0003 (0.0002)	0.3102	-0.0003 (0.0003)	0.2479
50-59	1037	-0.0004 (0.0003)	0.1475	-0.0007 (0.0003)	**0.0300**
60-69	1049	-0.0009 (0.0004)	**0.0210**	-0.0013 (0.0003)	**0.0016**
		**Former and Current**			
		**Crude**		**Adjusted[1]**	
**Age, y**	**N**	**β (SE)**	**p-value**	**β (SE)**	**p-value**
Unstratified	2006	0.0000 (0.0001)	0.9746	-0.0004 (0.0001)	**0.0041**
30-39	463	0.0003 (0.0003)	0.3524	-0.0001 (0.0003)	0.8310
40-49	500	0.0001 (0.0003)	0.5886	-0.0003 (0.0003)	0.2967
50-59	418	-0.0003 (0.0003)	0.2183	-0.0005 (0.0003)	0.1523
60-69	525	-0.0007 (0.0004)	0.0527	-0.0009 (0.0004)	**0.0329**
		**Current only**			
		**Crude**		**Adjusted[1]**	
**Age, y**	**N**	**β (SE)**	**p-value**	**β (SE)**	**p-value**
Unstratified	1033	-0.0005 (0.0002)	**0.0109**	-0.0005 (0.0002)	**0.0282**
30-39	296	-0.0003 (0.0004)	0.4959	-0.0004 (0.0005)	0.4311
40-49	307	-0.0004 (0.0004)	0.3476	-0.0007 (0.0004)	0.1150
50-59	246	0.0001 (0.0004)	0.7781	0.0001 (0.0004)	0.8154
60-69	184	-0.0013 (0.0006)	**0.0342**	-0.0016 (0.0004)	**0.0023**

Inverse Simpson index

		**Never, Former and Current**			

		Crude		Adjusted[1]	
**Age, y**	**N**	**β (SE)**	**p-value**	**β (SE)**	**p-value**
Unstratified	4387	0.0000 (0.0000)	0.7018	0.0000 (0.0000)	0.2337
30-39	1138	0.0000 (0.0000)	0.3199	0.0000 (0.0000)	0.6328
40-49	1163	0.0000 (0.0000)	**0.0272**	0.0000 (0.0000)	0.3157
50-59	1037	0.0000 (0.0000)	0.3736	0.0000 (0.0000)	0.1825
60-69	1049	-0.0001 (0.0000)	0.1968	-0.0001 (0.0000)	0.0580

		Former and Current			
		**Crude**		**Adjusted[1]**	
**Age, y**	**N**	**β (SE)**	**p-value**	**β (SE)**	**p-value**
Unstratified	2006	0.0000 (0.0000)	0.7532	0.0000 (0.0000)	0.1792
30-39	463	0.0000 (0.0000)	0.6134	0.0000 (0.0000)	0.8898
40-49	500	0.0000 (0.0000)	0.3087	0.0000 (0.0000)	0.9350
50-59	418	0.0000 (0.0000)	0.3354	0.0000 (0.0000)	0.3491
60-69	525	-0.0001 (0.0000)	0.1734	-0.0001 (0.0000)	0.1544

		Current only			
		**Crude**		**Adjusted[1]**	
**Age, y**	**N**	**β (SE)**	**p-value**	**β (SE)**	**p-value**
Unstratified	1033	0.0000 (0.0000)	0.2455	0.0000 (0.0000)	0.1527
30-39	296	0.0000 (0.0000)	0.8419	0.0000 (0.0000)	0.7208
40-49	307	0.0000 (0.0000)	0.2724	0.0000 (0.0000)	0.0936
50-59	246	0.0000 (0.0000)	0.6318	0.0000 (0.0000)	0.9494
60-69	184	-0.0001 (0.0001)	0.1970	-0.0001 (0.0000)	**0.0233**

Values are presented as beta (standard error, SE). N is the number of subjects for each stratum.
[1]Adjusted for age, sex, race/ethnicity, education, family income (PIR), BMI, alcohol consumption, physical activity, diabetes mellitus, hypertension, dyslipidemia and periodontitis.
