# Supplementary material for: Change of oral microbiome diversity by smoking across different age groups
Source: Front Microbiol. 2025 Dec 19;16:1714229. doi: 10.3389/fmicb.2025.1714229 (PMC12758414; doi:10.3389/fmicb.2025.1714229)
Supplement: Supplementary file 4 [file Data_Sheet_4.pdf]

Table S2. Concordance between linear-model and DESeq2 genus-level results.

taxa	short_tax	lm_direction	lm_estimate	lm_pvalue	baseMean	log2FoldChange	lfcSE	stat
RB_genus1062	Proteobacteria...Neisseria	Negative	-7.13E-05	2.09E-11	2073.314776	-0.006175751	2.79E-04	-22.11671405
RB_genus1232	Proteobacteria...Haemophilus	Negative	-8.45E-05	2.02E-09	4510.302334	-0.002716334	2.10E-04	-12.93347045
RB_genus475	Firmicutes...Gemella	Negative	-6.83E-05	6.02E-09	3843.851698	-0.002063998	1.90E-04	-10.8362496
RB_genus1227	Proteobacteria...Actinobacillus	Negative	-1.08E-05	9.65E-09	408.4093019	-0.008407682	0.002155301	-3.900932158
RB_genus499	Firmicutes...Abiotrophia	Negative	-4.12E-06	4.38E-08	137.4861713	-0.004991406	0.001228428	-4.063247595
RB_genus1160	Proteobacteria...Cardiobacterium	Negative	-1.06E-06	4.67E-08	33.77351739	-0.00475269	3.24E-04	-14.68318994
RB_genus318	Bacteroidetes...Bergeyella	Negative	-8.65E-06	1.22E-07	299.8762368	-0.004214315	2.56E-04	-16.44634327
RB_genus1228	Proteobacteria...Aggregatibacter	Negative	-1.03E-05	1.44E-07	353.224175	-0.005772768	0.001570226	-3.676392186
RB_genus644	Firmicutes...Peptococcus	Negative	-1.30E-06	2.12E-07	34.38797675	-0.00336837	3.60E-04	-9.352593285
RB_genus992	Proteobacteria...Lautropia	Negative	-2.86E-06	2.83E-07	86.42835914	-0.005851255	3.90E-04	-15.01202121
RB_genus650	Firmicutes...Peptostreptococcus	Negative	-6.92E-06	5.50E-07	191.5185642	-0.003631185	2.68E-04	-13.57142639
RB_genus39	Actinobacteria..Actinomycetaceae	Negative	-9.36E-07	8.43E-06	26.73938224	-0.004766848	3.42E-04	-13.94892118
RB_genus319	Bacteroidetes...Capnocytophaga	Negative	-5.10E-06	2.20E-05	214.8510259	-0.002457687	2.10E-04	-11.71028161
RB_genus626	Firmicutes..Lachnospiraceae	Negative	-2.89E-06	3.15E-05	123.4279778	-0.002889884	3.77E-04	-7.661162157
RB_genus256	Bacteroidetes...Alloprevotella	Negative	-2.15E-05	3.93E-05	1282.65909	-0.001632705	2.02E-04	-8.084750302
RB_genus583	Firmicutes...[Eubacterium]_nodatum_group	Negative	-2.69E-06	4.69E-05	117.4508713	-0.002380192	2.37E-04	-10.02548474
RB_genus379	Candidate_division_SR1	Negative	-3.17E-06	1.34E-04	71.60711201	-0.00465051	0.001372263	-3.388933912
RB_genus603	Firmicutes...Johnsonella	Negative	-5.72E-07	2.00E-04	13.14889185	-0.004627608	5.82E-04	-7.950739493
RB_genus261	Bacteroidetes...Prevotella_2	Negative	-2.07E-06	3.04E-04	91.79300156	-0.002356821	2.46E-04	-9.599045987
RB_genus1234	Proteobacteria..Pasteurellaceae	Negative	-2.49E-06	4.65E-04	67.6215517	-7.19E-04	0.004534785	-0.158490138
RB_genus698	Firmicutes...Bulleidia	Positive	1.67E-06	6.21E-07	24.56500821	0.003991673	5.52E-04	7.22475809
RB_genus135	Actinobacteria...Rothia	Positive	1.11E-04	4.89E-06	11492.12813	0.002319673	1.88E-04	12.34266267
RB_genus193	Actinobacteria...Atopobium	Positive	1.24E-05	1.96E-05	475.1734238	0.00292539	1.95E-04	15.00774186
RB_genus519	Firmicutes...Lactobacillus	Positive	6.13E-05	1.10E-04	861.5638409	0.010678738	0.003153377	3.386444606
RB_genus582	Firmicutes...[Eubacterium]_brachy_group	Positive	3.73E-06	1.74E-04	71.66789718	0.002523757	3.11E-04	8.105060923
RB_genus51	Actinobacteria...Scardovia	Positive	3.82E-06	1.81E-04	99.85420444	0.002147763	0.001452797	1.478364191
RB_genus49	Actinobacteria...Parascardovia	Positive	1.64E-06	3.71E-04	19.72730259	0.007523135	8.72E-04	8.631779397
RB_genus731	Firmicutes...Megasphaera	Positive	7.74E-06	5.18E-04	395.3630772	0.002296751	2.43E-04	9.439884019
RB_genus579	Firmicutes...Incertae_Sedis	Positive	1.59E-06	5.40E-04	26.48228762	0.003236055	6.45E-04	5.01544839

Text summary: This table lists te 29 genera significant in fully adusted linear models, alongisde

deseq_pvalue	deseq_padj	dsq_direction	direction_match
2.18E-108	1.51E-106	Negative	Match
2.91E-38	2.23E-37	Negative	Match
2.32E-27	1.23E-26	Negative	Match
NA	NA	Negative	Match
NA	NA	Negative	Match
8.26E-49	1.14E-47	Negative	Match
8.91E-61	3.07E-59	Negative	Match
2.37E-04	3.63E-04	Negative	Match
8.55E-21	2.95E-20	Negative	Match
6.13E-51	1.13E-49	Negative	Match
5.92E-42	5.10E-41	Negative	Match
3.19E-44	3.15E-43	Negative	Match
1.13E-31	7.08E-31	Negative	Match
1.84E-14	4.89E-14	Negative	Match
6.23E-16	1.79E-15	Negative	Match
1.18E-23	5.42E-23	Negative	Match
NA	NA	Negative	Match
1.85E-15	5.12E-15	Negative	Match
8.07E-22	3.27E-21	Negative	Match
NA	NA	Negative	Match
5.02E-13	1.28E-12	Positive	Match
5.34E-35	3.68E-34	Positive	Match
6.53E-51	1.13E-49	Positive	Match
NA	NA	Positive	Match
5.27E-16	1.58E-15	Positive	Match
NA	NA	Positive	Match
6.04E-18	1.89E-17	Positive	Match
3.73E-21	1.36E-20	Positive	Match
5.29E-07	1.07E-06	Positive	Match

: DESeq2 estimates on raw counts (baseMean, log2 fold-change, SE, Wald p-value, FDR), the DI

ESeq2 direction, and a direction-match indicator. All 29 genera showed concordant effect direc

tions; 24/29 (83%) were also significant by DESeq2 at FDR<0.05.
